# Comparative Sulcal Morphology of the Late Miocene Fossil Ape, *Rudapithecus hungaricus*

**DOI:** 10.1002/ajpa.70218

**Published:** 2026-02-23

**Authors:** Griffin A. Assance, Mary T. Silcox, David R. Begun

**Affiliations:** ^1^ Department of Anthropology University of Toronto Toronto Ontario Canada; ^2^ Department of Anthropology University of Toronto Scarborough Scarborough Ontario Canada

**Keywords:** brain evolution, encephalization, endocasts, hominids, hominines

## Abstract

**Objectives:**

Endocasts of fossil hominoids are exceedingly rare. The only fossil ape endocast analyzed in detail is that of *Ekembo nyanzae* (KNM‐RU 7290), from the early Miocene of Kenya. Two partial crania of *Rudapithecus hungaricus*, from the late Miocene of Hungary, preserve sufficient details to reconstruct large amounts of endocranial morphology of this fossil hominine. No other non‐hominin fossil hominid endocast preserves external morphology with the same completeness and detail. Here, we provide the first complete description of the *Rudapithecus* sulcal pattern.

**Materials and Methods:**

Sulcal patterns from a comparative sample of extant non‐hominin hominoid endocasts were identified according to classical descriptions of hominoid sulcal patterns. Sulcal patterns of both *Rudapithecus* endocasts were identified using both classical descriptions of hominoid sulcal patterns and comparisons with morphology identified in the extant sample.

**Results:**

We identify a hominid‐like sulcal pattern in *Rudapithecus* that is more complex than hylobatids but simpler than *Pan*, most closely resembling *Gorilla* and *Pongo* while exhibiting several *Gorilla* affinities.

**Discussion:**

The study of the *Rudapithecus* crania reveals a de‐coupling in hominid brain evolution. Cranial shape overall is African ape‐like, the shape of the endocast is more primitive, and the sulcal pattern is generally hominid‐like. The *Rudapithecus* endocasts provide evidence of the primitive condition of the brain in hominine evolution.

## 1 Introduction

The study of catarrhine brain evolution, and by extension, behavioral and cognitive evolution is complicated by the extreme rarity of relatively complete and well preserved endocasts and the intrinsic difficulty of extrapolating brain surface morphology from endocasts (Alatorre‐Warren et al. 2019; Begun 2024; Radinsky 1972; Tobias 1967; Phillip V. Tobias 1987). Recently Dumoncel et al. (2021) provided support for endocasts as reliable proxies for brain external organizational morphology in fossil taxa. With this in mind, we present the first comprehensive analysis of two partial endocasts of the fossil great ape *Rudapithecus hungaricus. Rudapithecus* fills a gap in the fossil record of brain evolution between *Ekembo* and *Australopithecus*, revealing dynamics of brain evolution previously unknown for the Hominoidea (gibbons, great apes, humans, and their fossil relatives).

## 2 Background

Radinsky (1974, 1975) ushered in the modern era of primate paleoneurology with his many studies of fossil mammal endocasts (Radinsky 1972, 1973, 1974, 1975). Among them were his analyses of the endocranial morphology of *Aegyptopithecus zeuxis*, an Oligocene stem catarrhine, and *Ekembo nyanzae* (then *Dryopithecus africanus*, later *Proconsul africanus*), an early Miocene stem hominoid (Radinsky 1973, 1974, 1975). In his analysis of the *Aegyptopithecus* sulcal pattern, Radinsky identified a transverse central sulcus that indicates clear catarrhine affinities, as most modern strepsirrhines instead display a longitudinal coronal sulcus (Radinsky 1973, 1974, 1975). On the *Ekembo* endocast (KNM‐RU 7290, formerly BMNH 32363), Radinsky (1974) identified a sulcus rectus (*r*; see Table 1 for sulci abbreviations), central sulcus (*c*), horizontal intraparietal sulcus (*ip*), superior precentral sulcus (*pcs*), subcentral anterior sulcus (*sca*), a sylvian fissure (*s*), and a superior temporal sulcus (*ts*). Radinsky (1974) concluded that the sulcal pattern of *Ekembo* did not differ significantly (aside from the absence of a superior frontal sulcus) from extant hominoids, likening the *Ekembo* pattern to that of hylobatids. Radinsky's (1974) preliminary estimate of cranial capacity (based on comparisons with extant baboons) was also in line with what would be expected for a primitive hominoid at 150 cc (about the size of a baboon, matching its estimated body mass; Begun and Kordos 2004). The *Ekembo* endocast was later re‐evaluated by Falk (1983), who did not provide an estimation due to the significant distortion and fragmentary nature of the fossil, but agreed that the size of the endocast is baboon‐like. She also identified many more sulcal impressions, particularly in the parietal lobe, such as the anterior branch of the superior temporal sulcus (*a* ^*1*^), the descending branch of the superior temporal sulcus (*a* ^*3*^), postcentral superior sulcus (*pts*), and the lunate sulcus (*L*). Additionally, she noted the conspicuous absence of a superior frontal sulcus (*fs*) and arcuate sulcus (*arc*) (among others), which is notable as *fs* and *arc* are present in most catarrhines (Falk 1983). Falk cited several missing key sulci (i.e., *fs*, *arc*, *io*, *fo*) as evidence that the *Ekembo* endocast is more primitive than Radinsky believed, although she interpreted the presence of the newly identified *a* ^*1*^ and *a* ^*3*^ as evidence of shared derived traits with catarrhines and hominoids, respectively. However, Falk (1983) also concluded that the *Ekembo* endocast was both too complex in sulcal patterning and too large in cranial capacity to be ancestral to hylobatids, while not preserving any derived sulci shared exclusively with hominids (Hominidae; great apes, humans, and their fossil relatives) that suggest an ancestor–descendant relationship. Additionally, several regression‐based estimates of cranial capacity in *Ekembo* have been published using its cranial midline arc (167.3 cm^3^; Walker et al. 1983) and foramen magnum area (130.3 cm^3^; Manser and Harrison 1999), providing further evidence for a papionin‐like cranial capacity in *Ekembo* (despite lingering uncertainty surrounding the fossil's preservation; Begun and Kordos 2004). The goal of this paper is to partially bridge the large paleoneurological gap between *Ekembo* in the early Miocene and the earliest *Australopithecus* endocasts in the Pliocene (Beaudet et al. 2018, 2019; Falk 1985, 1987; Granger et al. 2022; Holloway et al. 2004; Holloway and Yuan 2004) with an analysis of two endocasts of the late Miocene hominine (Homininae: African apes, humans, and their fossil relatives) *Rudapithecus* from Rudabánya, Hungary, dated to about 10 Ma (Kordos and Begun 2002).

**TABLE 1:** Sulcal abbreviations used here.

Abbreviations	Corresponding sulcus
a^1^/a	Anterior branch of the superior temporal sulcus
a^2^	Middle branch of the superior temporal sulcus
a^3^	Descending branch of the superior temporal sulcus
arc	Arcuate sulcus
b	Sublunate sulcus
c	Central sulcus
cm	Callosomarginal sulcus
d	Diagonal sulcus
e	Processus acuminus
fi	Inferior frontal sulcus
fm	Midfrontal sulcus
fo	Fronto‐orbital sulcus
fs	Superior frontal sulcus
h	Horizontal branch of the arcuate sulcus
io	Opercular sulcus
ip	Horizontal intraparietal sulcus
L	Lunate sulcus
lb	lambdoid suture
lc	Lateral calcarine sulcus
o	Orbital sulcus
oci	Inferior occipital sulcus
oct	Occipito‐temporal sulcus
pci	Inferior precentral sulcus
pcs	Precentral superior sulcus
pm	Paramedial sulcus
po	Parieto‐occipital sulcus
ps	Superior parietal sulcus
pti	Postcentral inferior sulcus
pts	Superior postcentral sulcus
r	Sulcus rectus
rc	Retrocalcarine sulcus
s	Sylvian fissure
sca	Subcentral anterior sulcus
scp	Subcentral posterior sulcus
tm	Midtemporal sulcus
ts	Superior temporal sulcus
u	Upper branch of the lateral calcarine sulcus
W	Fronto‐marginal of Wernicke

*Note:* All sulci names and abbreviations are taken from Connolly (1950). However, since *arc* is not given a separate abbreviation by Connolly (1950), here we follow Falk (1983), where *arc* is defined as *pci* + *h*.

### 2.1 Rudapithecus

RUD 77^1^ is a partial cranium including a large section of the neurocranium, partial orbit and fragmentary maxilla, first described by Kordos (1987) and later with significant modifications and in more detail by Kordos and Begun (1997). The endocranial surface of RUD 77 is well‐preserved with a nearly complete right and partial left frontal lobe, a well‐preserved right parietal lobe, fragmentary left parietal lobe, and a partial occipital lobe (Kordos and Begun 1997). While the canines are not preserved, the morphology of the maxilla, the preserved portions of the canine alveolus, and its overall size identify RUD 77 clearly as a female (Begun and Kordos 2004; Kordos and Begun 1997). Six regressions based on cranial length used by Begun and Kordos (2004) estimate the cranial capacity range of RUD 77 to be between 302 and 350 cc with an encephalization quotient (EQ) of 2–2.35, within range of modern great apes and even some australopithecines (Jerison 1973; Kordos and Begun 1998; Martin 1981).

RUD 200 is a well‐preserved skull first described by Kordos and Begun (2001). Following the recovery of the associated mandible, it was subsequently virtually reconstructed and described in more detail in Gunz et al. (2020). RUD 200 preserves nearly complete frontal and right parietal bones, with portions of the occipital and temporal bones also preserved, as well as much of the face and mandible (Kordos and Begun 2001). RUD 200 is among the smallest *Rudapithecus* specimens, and it preserves all four canines, leaving no doubt that it is female. Gunz et al. (2020) reconstruction and Three‐Dimensional Geometric Morphometric (3DGM) analysis involved the virtual reconstruction of the skull, which differs from the reconstruction of the original specimen, in particular with respect to the retro‐deformation of frontal near glabella, and in its extensive mirroring imaging (Gunz et al. 2020: figure 6). Gunz et al. (2020: figures 11–13) found that while the 3DGM analysis of the external skull aligns it with African apes, the shape of the endocast falls closest to that observed in hylobatids. The various reconstructions of RUD 200 have yielded different estimates of cranial capacity at 280–330 cc (based on six cranial length regressions; Begun and Kordos 2004) and 221–247 cc (landmark‐based estimate using a three‐dimensional geometric morphometric reconstruction of the endocast; Gunz et al. 2020). The smaller estimates still fall within the range of extant *Pan* females, which are otherwise cranially, dentally and postcranially larger than RUD 200 (Begun and Kordos 2004; Gunz et al. 2020; Ward et al. 2019). This suggests a combination of a great ape‐like encephalization and relatively more primitive endocranial morphology in *Rudapithecus*, as represented by 3DGM (Gunz et al. 2020: figure 13). However, that analysis did not take sulcal patterns into consideration.

Unlike the *Ekembo* endocast, the *Rudapithecus* endocasts' sulcal patterns, proportions, and symmetries are minimally described. Kordos and Begun (2001) note the well‐preserved sulcal impressions on the endocranial surface of RUD 200 without further comment. Begun and Kordos (2004) described the shape of the endocast as being asymmetric and broader transversely than *Ekembo* and *Hylobates*. They also briefly touched upon the sulcal impressions present on both endocasts of *Rudapithecus*, with a clear and complex sulcal pattern present on the frontal lobe (Begun and Kordos 2004). Begun and Kordos (2004) describe a well‐defined *r*, *arc*, and *c* present on both *Rudapithecus* endocasts, with two other clear impressions that they surmise are likely *pcs* and *fs*. In the current paper, the sulci present on the endocasts of both RUD 77 and RUD 200 are fully described and compared to the patterns in a comparative sample of virtual hominoid endocasts.

## 3 Materials and Methods

### 3.1 Data Acquisition

**FIGURE 1:**
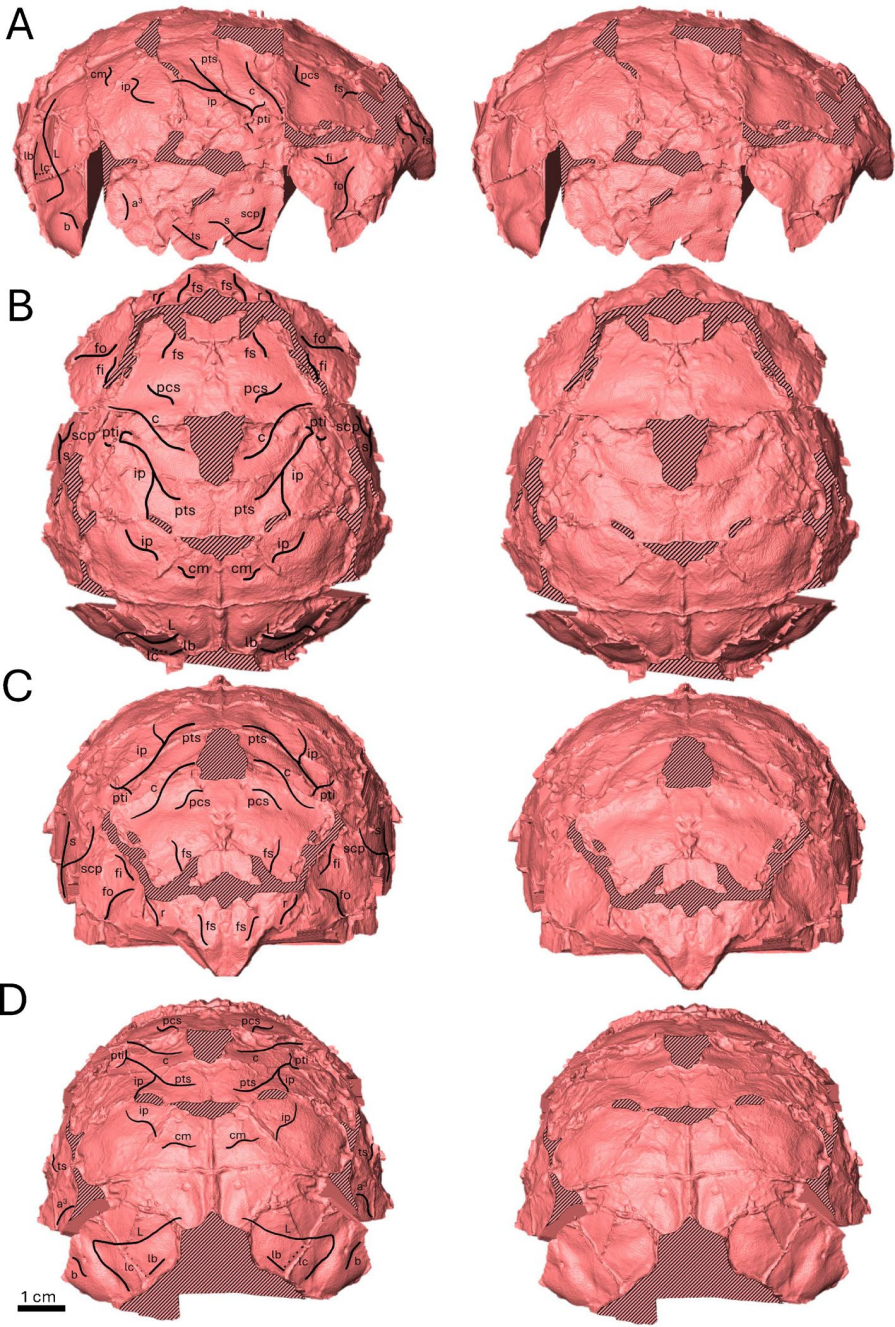
Female *Rudapithecus hungaricus* endocast (RUD 200; Gunz et al. 2020) with labeled sulcal pattern (left) with corresponding view unlabeled (right). (A) Lateral, (B) dorsal, (C) rostral, (D) Caudal. Gunz et al. (2020) reconstruction is mirrored in the sagittal plane.

**FIGURE 2:**
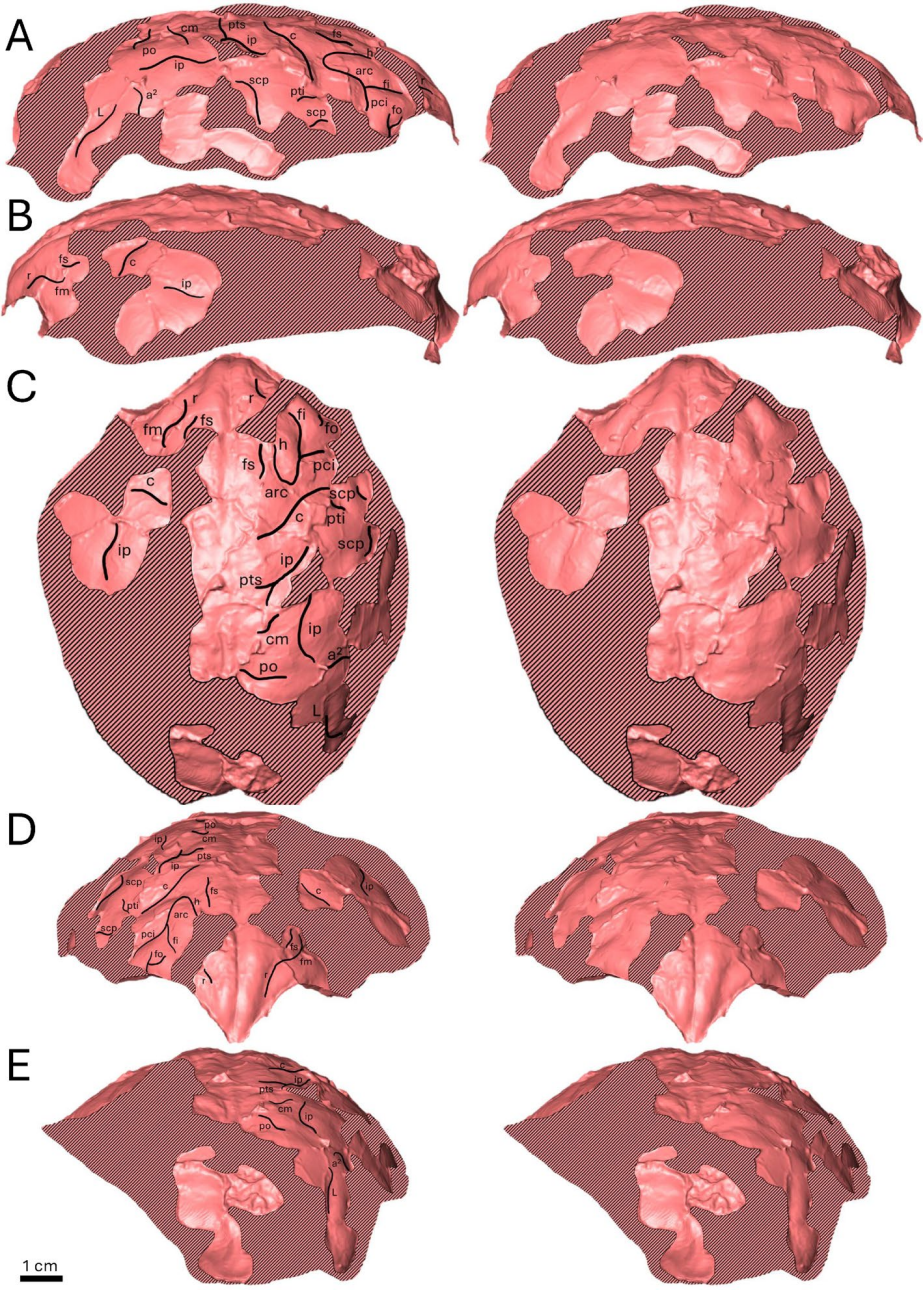
Female *Rudapithecus hungaricus* endocast (RUD 77) with labeled sulcal pattern (left) and corresponding view unlabelled (right). (A) Right lateral, (B) left lateral, (C) dorsal, (D) rostral, (E) caudal.

**FIGURE 3:**
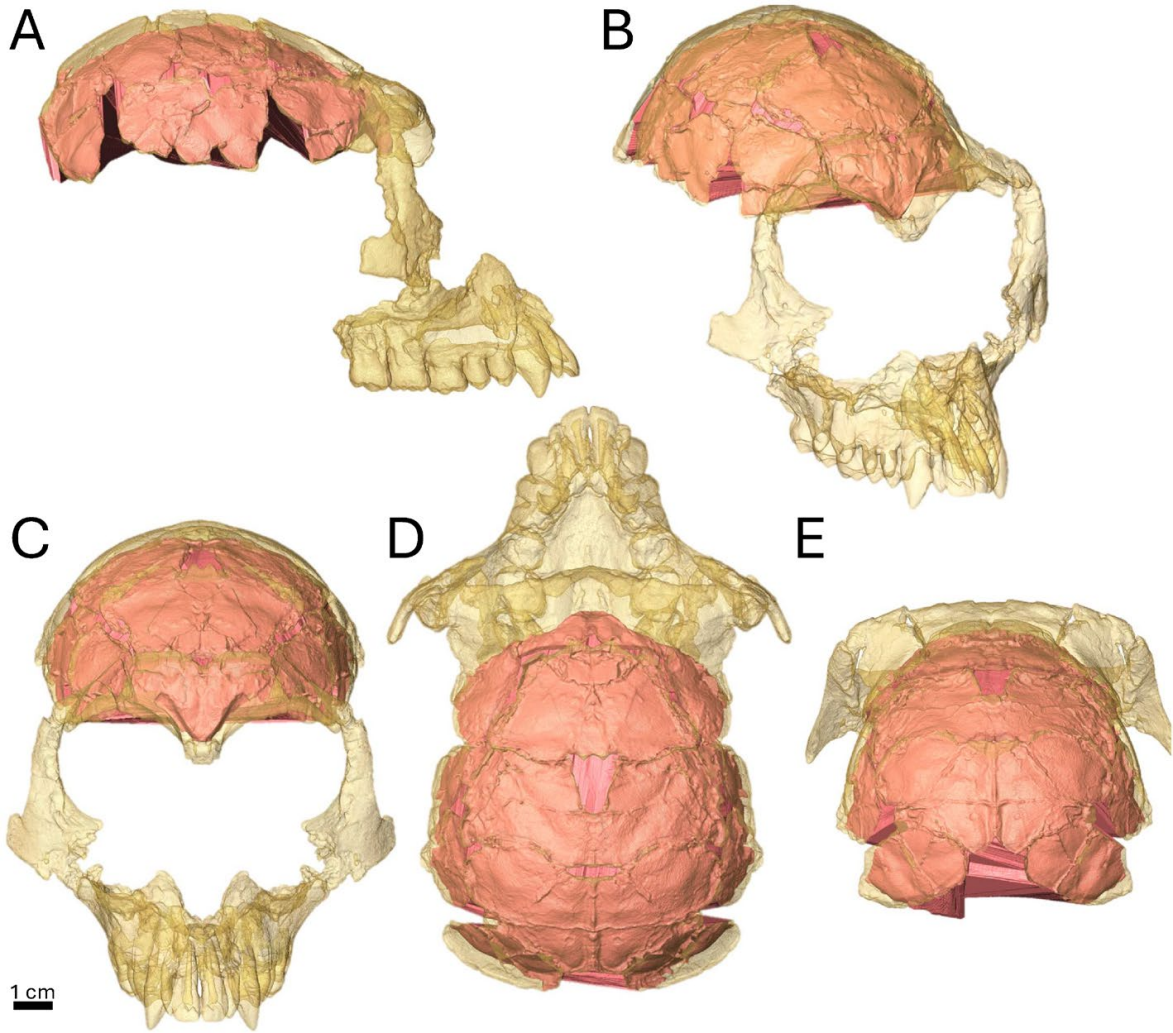
Gunz et al. (2020) reconstruction of the RUD 200 *Rudapithecus hungaricus* cranium (yellow) with virtual endocast (red). (A) Lateral, (B) oblique, (C) rostral, (D) dorsal, (E) caudal. Gunz et al. (2020) reconstruction is mirrored in the sagittal plane.

For RUD 200 (Figures 1 and 2), a 3D surface of the Gunz et al. (2020) reconstruction was converted from its original file type (.ply) to a TIFF stack using the “scan to volume” utility in Avizo 2023 to allow for segmentation of the endocranial space. The conversion makes the voxel size slightly larger (Table S1), but the resulting decrease in resolution for a specimen of this size does not significantly affect the quality of the endocast nor any identification of morphology that may be present on the surface. For RUD 77, the physical endocast (molded directly from the endocranial surface of the most recent reconstruction; Kordos and Begun 1997) was digitized and generated as a high‐resolution surface scan (Figure 3).

The comparative sample of endocasts consists of the crania from 10 individuals of each extant non‐hominin hominid taxon, including 5 males and 5 females of each taxon (Table S1). Additionally, a hylobatid outgroup consisting of 10 individuals of similarly sized hylobatid taxa (*Hylobates* and *Hoolock*; Reichard et al. 2016), also evenly divided into 5 males and 5 females (Table S1). Most individuals used in the comparative sample were acquired via MorphoSource (www.MorphoSource.org). To supplement the cranial data from MorphoSource, a sample of *Pongo* CT data consisting of five males and two female individuals was obtained from Dr. Matt Tocheri (Lakehead University; Table S1). For two of these *Pongo* scans, USNM 143588 and USNM 143594, the CT data was split into two parts that were not correctly registered to one another. To remedy this, the incorrectly registered portion in both datasets (the superior portion of the braincase) was rotated and then translated back into its proper orientation in Avizo 7.0, and the parts of the dataset were merged. Additionally, several individuals of the hylobatid sample (Table S1) that were procured from MorphoSource needed to be converted from a 3D surface (.ply) to a TIFF stack using the “scan to volume” utility in Avizo 2023 that also resulted in a slight increase in voxel size. For the larger TIFF stacks in the comparative sample only every other slice was included in the segmentation (doubling the voxel size in the *Z* dimension) to make the dataset size more manageable. This was accomplished by inputting the dataset into ImageJ (ImageJ.net) and re‐saving the tiff stack with only every other slice.

### 3.2 Endocast Scanning and Segmentation

Using a high‐resolution surface scanner (Gocator 3210A‐LED‐B‐20‐S), the physical RUD 77 endocast was digitized into a 3D surface file (.ply). The RUD 200 endocast was segmented utilizing a combination of Avizo 2023 and Avizo 7.0. For RUD 200, each CT slice was prepared using segmentation by closing any breaks in the superior surface of the cranium using a straight line and connecting the fossil's inferior‐lateral most margins. Next, using the prepared dataset, the endocranial surface was then segmented out, creating a rough virtual endocast. The surface of the virtual RUD 200 endocast is intentionally not smoothed or trimmed to the same extent as the other endocasts in the sample (Figure 1) as the fossil is significantly damaged, so over‐smoothing/cleaning the endocast risks losing data. Similar to RUD 200, the endocasts for the comparative sample were each segmented using the protocol outlined above, albeit only needing to close small breaks or foramina during the preparation stage as most of the crania present in the extant taxa were well‐preserved.

### 3.3 Sulcal Identification

The sulci present on the comparative endocasts were identified following Connolly (1950), which remains the standard reference for non‐hominin primate sulcal patterns (e.g., see Falk et al. 2018). Ensuring accuracy when identifying sulcal impressions on endocasts is a well documented challenge discussed in the paleoneurological literature (e.g., Falk et al. 2018; Labra et al. 2024; Neubauer 2014; Radinsky 1972; Tobias 1967; Tobias 1987) and was a significant focus here. To maximize identification accuracy and control for variation, the identification process was the same for each specimen. First, the frontal lobe was examined, beginning with matching prominent sulci to anatomically appropriate impressions in the endocast (i.e., *c*, *arc*, *r*, *fs*). Once prominent sulci were identified on the endocast, lesser sulci were identified (i.e., *fo*, *o*, *W*, *pcs*, *sca*, etc.…) based on their spatial relationship with previously identified sulci, as shown in Connolly (1950). This process of sulcal identification was then repeated for each lobe present on the comparative endocasts. Significant inter‐individual variation exists in sulcal patterning (Falk et al. 2018; Rademacher et al. 1993; Ribas 2010) and factors that influence the preservation of sulcal impressions in endocasts (e.g., thickness of the meninges, proportion of cerebrospinal fluid in the endocranial cavity, the fit of the brain in the endocranial cavity; Neubauer 2014), leading to endocranial morphology varying widely between individuals and taxa. Accordingly, inter‐individual variation was also observed here, with sulcal impressions in the comparative sample differing slightly in shape and spatial positioning among individuals (as in modern Human endocasts; de Jager et al. 2019).

The analysis of the *Rudapithecus* endocasts was conducted as described above but was complicated due to damage to the endocranial surface in both fossils, resulting in incompletely preserved or obscured impressions. In these cases, the spatial relationship of sulci demonstrated in Connolly (1950) aided in identifying the continuity of sulci and the relationship of incomplete sulci to other sulci surrounding the damage. Additionally, since the Gunz et al. (2020) reconstruction of RUD 200 utilized mirroring in the sagittal plane, only one hemisphere in RUD 200 was analyzed to minimize redundancy (see Figures 1 and 2). All sulci identified in this study are listed in Table S2.

## 4 Results

### 4.1 Sulcal Pattern of *Rudapithecus hungaricus*

#### 4.1.1 RUD 77 Endocast

##### 4.1.1.1 Frontal Lobe

There are five clear sulcal impressions on the right side of the frontal lobe of the RUD 77 endocast (Figure 3; see Table S2). At the rostral tip of the endocast, a short sulcus rectus (*r*) with a slight dorsal‐facing curve is present before a break in the endocranial surface interrupts the continuity of the sulcus. Just caudal to the break is another continuous longitudinal sulcus which terminates caudally at an adjacent perpendicular sulcus. Rostrally, this longitudinal sulcus is likely a continuation of *r*. Caudally, the position and termination of this impression is more like the inferior frontal sulcus (*fi*) but, for *Gorilla* and *Pan*, has been previously shown to be similarly located and connected to a perpendicular transverse sulcus (Connolly 1950: figures 72, 75, 80). It is unclear if the sulcus in question includes a continuation of the caudal portion of *r* or is a well‐defined *fi*; to be conservative, here it is defined as *fi* as the break in the endocranial surface is substantial, making determining the *r* caudal termination guesswork at best (Figure 3). The perpendicular sulcus at the caudal termination of *fi* is a very well‐defined arcuate sulcus (*arc*). Hereafter, we refer to *arc* as it is defined by Falk (1983), rather than following Connolly (1950), where *arc* is differentiated into two distinct sulci (*h* and *pci*). A definition of the arcuate sulcus comprised of discrete sections (as in Connolly 1950) has recently been re‐proposed (Amiez et al. 2023). However, for the present study, the overall form of the arcuate sulcus is clearly distinguishable from neighboring sulci in catarrhines that exhibit *arc* and, as such, the single sulcus definition (i.e., “*arc*”) is more appropriate for this study. The shape of *arc* on RUD 77 is very distinct. The origin of the horizontal branch of *arc* (*h*) is caudal to the aforementioned break and dorsal to *fi*, and remains longitudinal before curving ventrally, displaying a slight rostral curvature. It then settles into a transverse orientation and terminates inferior to the termination of *fi*, with the precentral inferior sulcus (*pci*) at a break at the lateral edge of the endocast creating a large, hooked shape sulcus with a stem inferior to the *fi* termination (Figure 3). The superior frontal sulcus (*fs*) is located superior to the horizontal branch of *arc*, on the dorsal‐most portion of the endocast, and runs longitudinally with a slight medial flare at the caudal termination. Finally, rostral to the inferior termination of *arc* and inferior to *fi* is a small fronto‐orbital sulcus (*fo*) faintly visible at the endocast's edge, which originates as a stem oriented transversely before forking into two short arms, one continuing dorsally and another pointed rostrally with a slight dorsal curve (Figure 3).

**FIGURE 4:**
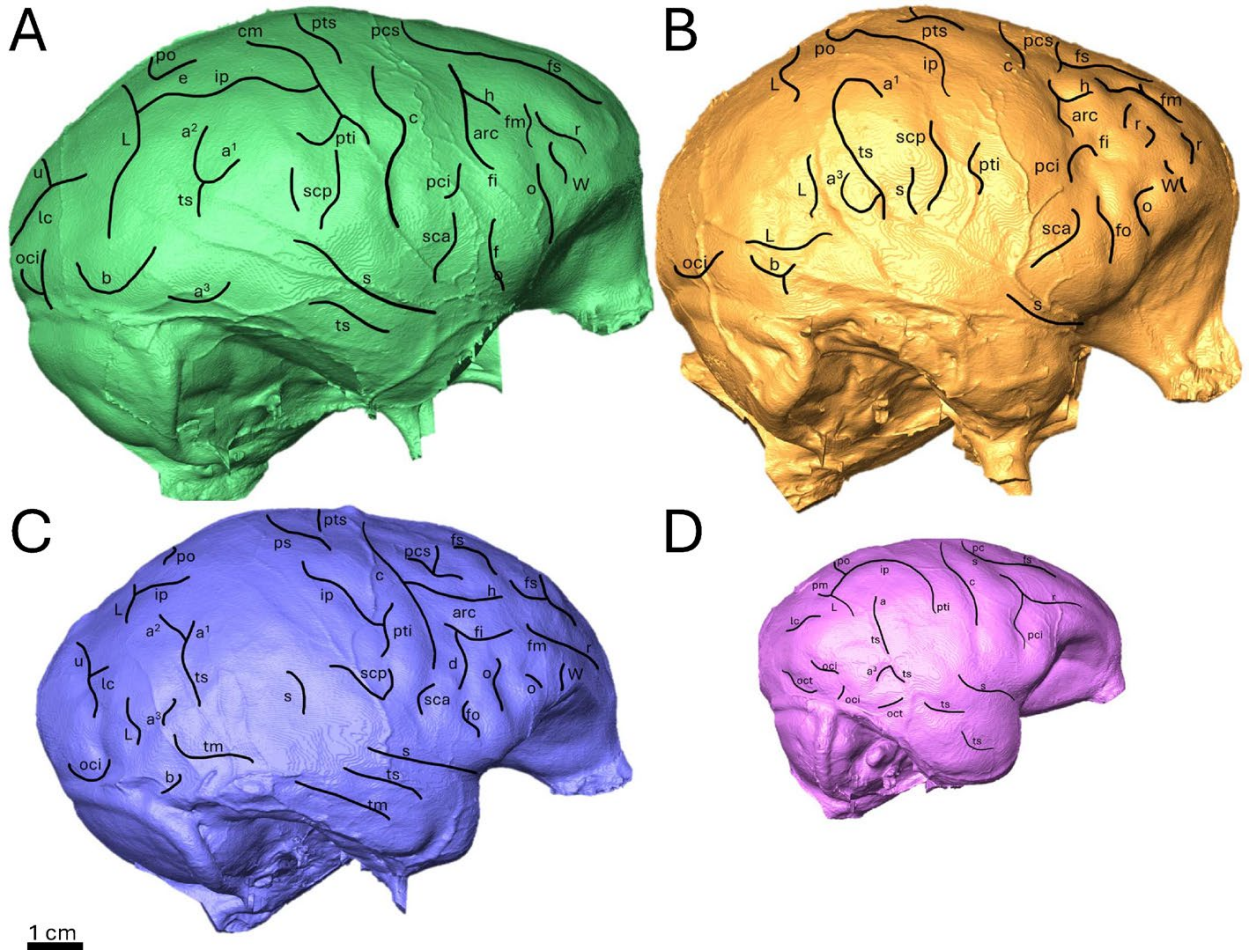
Example set of extant hominoid endocasts with labeled sulcal patterns, see Supporting Information S1 for the full extant comparative sample used here. (A) *Gorilla* (Female, MCZ 37265), (B) *Pongo* (Female, DMNS 1064), (C) *Pan* (Female, MCZ 17702), (D) *Hylobates* (Female, MCZ 41440).

On the left side of the frontal lobe the sulci are fainter, but some are still recoverable, with two distinct discernable impressions. The first impression is much like that observed rostrally on the right frontal lobe, with an obvious sulcus rectus (*r*) nearly mirroring the same sulcus on the other side (Figure 3). However, no break is present on the left side, meaning that the sulcal impression continues longitudinally past what could reasonably be considered *r*. From the spatial positioning of this impression, it was determined that the caudal portion of this impression likely includes the origin of a midfrontal sulcus (*fm*) (Figure 3), a relationship that was noted as a variation in these sulci in all hominids by Connolly (1950: figures 56, 76, 82; see also Falk et al. 2018: figures S1–S4, S6, S8; Figures 4C, S1, S19, S24 here). The other sulcal impression is a short, longitudinally running superior frontal sulcus (*fs*), which runs dorsal to the aforementioned impression of *r* + *fm* before terminating after a slight medial flare.

##### 4.1.1.2 Parietal Lobe

There are 11 parietal sulci present on the endocast of RUD 77 (Figure 3; Table S2): nine on the more complete right side and two on the fragmentary left side. Of the nine sulci, four are located on the superior portion of the right parietal lobe. A prominent central sulcus (*c*) originates just lateral to the sagittal midline before running transversely, curving rostrally before terminating in‐line longitudinally with *fi*, medial to a significant break in the endocast. Caudal to *c* there are two connected sulcal impressions, one flaring dorsally nearly parallel to *c* connecting inferiorly to the other that runs somewhat longitudinally with a lateral curve (Figure 3). The dorsal‐most section is the superior postcentral sulcus (*pts*), which was identified due to its spatial position and orientation in relation to *c* in addition to its connection with the other sulcus, the longitudinally running horizontal intraparietal sulcus (*ip*). *ip* is one of the longest sulci present on the endocast of RUD 77. However, there are two distinct portions that are unconnected but spatially appropriate to be considered the same sulcus: the aforementioned rostral portion and a longer, caudal portion. The caudal portion of *ip* originates at a crack in the endocranial surface towards the caudal end of the endocast before running longitudinally, curving dorsally and then terminating in‐line transversely with a spur at the junction of the rostral portion of *ip* and *pts* (Figure 3). Dorsal to the caudal portion of *ip* is a small transverse callosomarginal sulcus (*cm*) which, like *pts* and *c*, originates near the sagittal midline before flaring rostrally. Inferior to the caudal‐most origin of *ip* is a single transverse sulcus with a caudal flare at the superior end. This sulcus is spatially appropriate and of the proper orientation in relation to *ip* to be the middle branch of the temporal superior sulcus (*a* ^*2*^). Such a spatial relationship between *a* ^*2*^ and *ip* is seen in many hominid brains (Connolly 1950: figures 60, 67, 84) and endocasts (e.g., Figures 4, S1, S12, S19).

On the inferior portion of the endocast there are five sulci. The first is immediately caudally inferior to *c* and is defined as a small postcentral inferior sulcus (*pti*) due to its perpendicular orientation to *c* and spatial relationship with *ip* (Figure 3). This relationship with *ip* is best explained as a nearly perpendicular intersection that would occur if the two sulci were to meet on the endocast. Such a relationship between *ip* and *pti* is observed in many hominid brains and endocasts (Figure 4A) (Connolly 1950: figures 53, 65, 67, 72, 80, 83, 84; figure 4). Two sulcal impressions are near *pti*. One is immediately inferior to *pti*, sharing its orientation with the caudal termination hidden behind the edge of the endocast. The other is positioned caudally at a nearly perpendicular angle to *pti*. It exhibits a caudal curvature on the dorsal‐most termination, with the inferior portion fading before reaching the edge of the endocast. Both impressions surrounding *pti* are likely branches of a large subcentral superior sulcus (*scp*) (Figure 3). Connolly (1950: figures 53, 54, 72, 76) has shown that in hominids, *scp* is inferior and caudal to *c* (too far superior to be the sylvian fissure in RUD 77; Figure 3), while also being a large sulcus that may have branches (also observed here; Figures 4, S3, S7, S23).

On the left parietal lobe, there are two distinct sulci, *c* and *ip*. The left *c* is located just caudal to *fs* on the opposite side of the crack in the endocranial surface. The left *c* is slightly more rostral than the *c* on the right side but oriented with a rostral curve much like the opposite side, similar enough in shape and location to be considered the corresponding sulcus. This slight variation in sulcal positioning between hemispheres was commonly observed in the comparative sample here and is therefore likely indicative of between‐hemisphere variation at the individual level rather than asymmetry in cortical organization. On the left side *ip* is a long longitudinal sulcus that is isolated from other discernable sulci (aside from *c*, located rostral to *ip* and oriented nearly perpendicularly). Since no other sulci were present to provide additional context, the shape and spatial position of the corresponding sulci (i.e., in the proper spatial position relative to *c*) on the right side were used to aid in identifying *ip* as the most likely identification for the second sulcus.

##### 4.1.1.3 Occipital Lobe

Only two sulci are present on what is preserved of the occipital lobe of RUD 77 (Figure 3E; see Table S2). One is a short transverse, rostrally curved parietooccipital sulcus (*po*) located just caudal to *cm* and the caudal‐most portion of *ip*, fading before reaching the longitudinal position of *ip*. Near the very caudal‐most portion of the endocast there is a prominent sulcus originating at the edge of the endocast, running longitudinally towards the sulcus identified as *a* ^*2*^. This sulcus is important as the shape, location, and prominence are similar to the lunate sulcus (*L*). The portion of *L* on RUD 77 is angled longitudinally (Figure 3E), which is found in some hominids (particularly in *Gorilla* and *Pongo*, less so in *Pan*) as a common shape for the inferior‐most termination of *L* in Connolly (1950: figures 62, 69) and this study (e.g., Figure 4B, see also Figures S2, S23, S25).

#### 4.1.2 RUD 200 Endocast

##### 4.1.2.1 Frontal Lobe

The frontal lobe on the endocast of RUD 200 preserves six sulcal impressions, four dorsally and two inferiorly (Figure 1; see Table S2). At the rostral tip of the endocast there are two impressions. The dorsal‐most is a small longitudinal *fs* with a slight medial curve at the rostral termination that is obscured caudally by a significant crack in the endocast. Another sulcus is present in the same orientation as the caudal termination of the rostral portion of *fs*. With the spatial positioning and orientation congruency of this impression with *fs*, it is very likely a continuation of *fs* beyond the aforementioned crack (Figure 1). The second impression at the rostral tip of the endocast is a short longitudinal *r* with a small medial bend at the rostral termination, located slightly inferior and caudal to the rostral portion of *fs* which, like *fs*, fades before the impression reaches the crack in the endocast. The final impression on the dorsal portion of the frontal lobe is a well‐defined superior precentral sulcus (*pcs*). *pcs* is caudal to the caudal portion of *fs* but runs longitudinally at the rostral end before curving at the caudal end to an orientation like the transverse orientation of the *c* dorsal termination, which is located just caudal to *pcs*.

About halfway between the ventral and dorsal aspects of the frontal lobe on RUD 200 there is a break obscuring the area of the endocranial surface where significant portions of *arc* are normally located in extant hominids (Connolly 1950: figures 54–56, 67, 73). Below this break there are two recoverable sulcal impressions. The first is a longitudinal *fi* with a slight ventral curve just below the break, unconnected to any sulcal impression that could be interpreted as *arc*. Inferior to *fi* is a well‐defined, transverse “S”‐shaped *fo* which, at the superior termination, curves caudally, then runs towards a slight break in the endocranial surface before curving back rostrally as the impression fades before reaching the edge of the endocast.

##### 4.1.2.2 Parietal Lobe

There are nine sulcal impressions present on the parietal lobe of RUD 200, six above a break running longitudinally across the middle of the lobe, and three below the break (Figure 1; see Table S2). Above the break, on the dorsal midline of the endocast, there is another extensive break in the endocranial surface. Lateral to the caudal end of the midline break and caudal to *pcs* on the frontal lobe is a well‐defined *c*, which is oriented transversely at its caudomedial origin but curves rostrally as the sulcus continues down the endocast. Caudal to the inferior termination of *c* is an intersection of two sulcal impressions. One is a longitudinal *ip* running across the parietal lobe and the other is very likely *pti*, despite an unusually dorsal location (Figure 1), since its intersection is nearly perpendicular to *ip* as is common in extant hominids (Connolly 1950: figures 53, 65, 82; Figures 4, S7, S18, S26 here). Dorsal to *ip* there is a small *pts* that curves caudally at its medial origin before running diagonally down the endocast rostrally and intersecting with *ip* at its inferior termination. Further, there is an “S” shaped impression caudal to the caudal termination of *ip* that is likely, as in RUD 77, a continuation of *ip* (Figure 1). While the shape of the impression is unexpected for the caudal portion of *ip*, this portion of the impression is likely a branch of *ip*. The shape does not resemble any other nearby sulci, nor does its location allow for any reasonable attribution to another sulcus (as *ip* would need to pass through that location, see Connolly 1950: figures 60, 65, 82). Additionally, caudal and medial to the branch in *ip* there is a small transverse *cm* that originates just lateral to the midline of the endocast.

Much like the frontal lobe of RUD 200, the parietal lobe is also separated by a significant break in the endocranial surface into superior and inferior portions. Below this break are three more sulcal impressions. The rostralmost sulcus on the inferior parietal lobe is a large, rostrally curved *scp* that points towards the rostral origin of the major parietal break at its superior termination and intersects the caudal end of the sylvian fissure (*s*) at its inferior termination (Figure 1). This impression is identified as *scp* for the same reasons as *scp* was identified in RUD 77, its location and shape being congruent with *scp* in extant hominids. Other evidence of its attribution as *scp* is the location of the intersection of this impression and *s*, being caudal relative to *c*. This positioning eliminates the subcentral anterior sulcus (*sca*) as a candidate (Connolly 1950: figures 54–56, 67, 72, 82, 84). *s* is a well‐defined sulcus on the inferior portion of the parietal lobe, discernable just above the edge of the endocast. It runs longitudinally from its rostral origin at the edge of the endocast, past its intersection with *scp*, before terminating after a slight inferior curve. The final sulcus on the parietal lobe of RUD 200 is a faint, rostrally curved impression of the descending branch of the temporal superior sulcus (*a* ^*3*^), which is located caudal to *s*, just rostral to the edge of the endocast (Figure 1). The attribution of *a* ^*3*^ to this impression is challenging, as the only nearby sulci aiding identification are the superior temporal sulcus (*ts*) and the sublunate sulcus (*b*). The location and orientation of the impression identified as *a* ^*3*^ relative to *ts* may be indicative of an intersection in the brain between these two sulci, which in extant hominid brains occurs at the superior end of *a* ^*3*^ (Connolly 1950: figures 67, 72, 83). Additionally, the impression identified as *a* ^*3*^ here is just caudal to *b*, which is normally adjacent or even sometimes, as in *Pongo*, connected to *a* ^*3*^ (Connolly 1950: figures 53, 65, 72, 84; e.g., Figure 4A,B, S7, S13, S25).

##### 4.1.2.3 Temporal and Occipital Lobes

There is only one sulcus preserved on the temporal lobe of RUD 200 (Figure 1; see Table S2), *ts*, which originates rostrally at the inferior edge of the endocast inferior to *s* before running alongside *s* longitudinally and fading shortly thereafter. This impression is attributed to *ts* due to its clear spatial relationship with *s*. As in many hominoids, *ts* runs alongside from the rostral tip of the temporal lobe to the *s* caudal termination in the parietal lobe (Connolly 1950).

On the occipital lobe three sulcal impressions are discernable, two well‐defined and one faint (Figure 1D; see Table S2). The most prominent is a large *L*, which is longitudinal at its most inferior portion and curves transversely as the sulcus extends towards the medial edge of the endocast (Figure 1D). The shape of this sulcus is much like the partial *L* recovered on RUD 77. The inferior longitudinal portion is sharply curved rostrally, leading to a termination angled towards the caudal tip of the occipital lobe in both the brains (Connolly 1950: figures 53, 62, 65, 69) and endocasts (e.g., Figure 4B, see also S1, S2, S23, S25) of *Gorilla* and *Pongo*. Inferior to *L*, is a short transverse *b* that runs alongside the transverse inferior portion of *L*. This sulcus is easy to identify due to its clear inferior relationship to *L*, running alongside *L* as is in extant hominid brains (Connolly 1950: figures 53, 72, 84). Lastly, caudal to the transverse portion of *L*, is a ridge that is likely a faint imprint on the endocast left by the lambdoidal suture (*lb*; see Falk et al. 2025: figure 6d). Intersecting *lb* at a nearly perpendicular angle, where the lateral calcarine sulcus (*lc*) is located in extant hominoids (Connolly 1950: figures 62, 69, 84), there is a faint longitudinal impression that could be *lc* (Figure 1D). This sulcus is faint but in the exact position where *lc* would normally be in extant hominids (Connolly 1950: e.g., figures 62, 69, 84). Given its poor preservation and to reflect some uncertainty, *lc* is labeled in Figure 1 with a dotted line to distinguish this faint sulcus from the other better‐defined sulci.

##### 4.1.2.4 Rudapithecus Compared With Extant Hominoids

There are many differences between the sulcal pattern on the *Rudapithecus* endocasts and that observed in hylobatids. A major difference is the presence of a full *arc* (h + pci; as defined by Falk 1983) on the frontal lobe of RUD 77, which is not observed in any of the hylobatid comparative endocasts here nor is an *arc* comprised of both *h* and *pci* observed in Connolly's (1950) description of the hylobatid sulcal pattern. Interestingly, while it is true that hylobatids do not exhibit a complete and separate *arc* like hominids, several workers (including Connolly 1950: figures 26, 28, 33) have noted that a separate, hook‐shaped arc is very common in cercopithecoids (e.g., Amiez et al. 2023; Falk 1983; Gonzales et al. 2015). Regardless of whether *arc* in hominids is a plesiomorphic trait shared with cercopithecoids (Gonzales et al. 2015), the presence of *arc* on RUD 77 readily distinguishes the sulcal pattern of *Rudapithecus* from that of hylobatids. Further, it is also notable that several sulci identified on the *Rudapithecus* endocasts (i.e., *fo*, *b*, *scp*) are not observed in any of the hylobatid endocasts in the comparative sample here despite *fo* and *scp* being identified in vivo (Connolly 1950: figures 41, 46). Connolly (1950: figures 43, 60, 65, 84) also shows that the dorsal most aspect of the ascending tract of the superior temporal sulcus is made up of a continuous sulcus (*a*) in hylobatids but is commonly split superiorly into an anterior (*a* ^*1*^) and middle (*a* ^*2*^) branch in extant hominids (Connolly 1950: figures 60, 67, 84; Figures 4, S1, S12, S19 here). The hominid morphology is observed as *a* ^*2*^ is present on RUD 77 (Figure 3). Despite being incomplete, the general sulcal pattern of *Rudapithecus* appears to be more complex than in hylobatids, much more closely resembling that in *Gorilla* and *Pongo*.

The shape of *c* in both RUD 77 and RUD 200 is unlike the distinct, curving *c* present in *Pan*, which is so pronounced that it may superficially intersect *arc* and appear as continuous impression in endocasts (e.g., Figures 4C, S15, S16 here; also observed in vivo, albeit rarely; Connolly 1950: figure 84; Falk et al. 2018: S7, see also figure S8). *c* in both *Rudapithecus* specimens has a clear rostral tilt as it travels down the endocast as in *Gorilla*/*Pongo* (Figures 1 and 3, see also Figures 4, S3, S23; Connolly 1950: figures 56, 57, 65, 73) while not exhibiting any of the exaggerated curvature that is much more common in *Pan* (Connolly 1950: figures 82, 84; Falk et al. 2018: figures S2, S3, S4, S6, S7, S8). The impression identified as the horizontal branch of *arc* in *Rudapithecus* (only visible in RUD 77; Figure 3) does not appear to intersect the curvature of *c*, but rather is part of a distinct “hook” shape that is common to the sulcal pattern of *Gorilla*/*Pongo* (Connolly 1950: figures 55–57, 65, 72; Figures 4A,B, S2–S4, S22, S23 here). Additionally, the inferior precentral sulcus (*pci* in Connolly 1950), which makes up the descending branch of *arc* as one continuous sulcus in *Gorilla*/*Pongo*, can be disconnected from *h* in *Pan* (Connolly 1950: figures 82, 84; Figures 4C, S12, S13), which is uncommon for *Gorilla* or *Pongo* (Connolly 1950: figures 55–57, 65, 72; Figures 4A,B, S2–S4, S22, S23 here). In RUD 77, where the entirety of *arc* is visible on the right side of the endocast, it has the continuous pattern of *Gorilla* and *Pongo* unlike *Pan. L*, like *arc*, easily distinguishes *Gorilla*/*Pongo* from *Pan*. As stated above, in *Pongo* and *Gorilla*, *L* takes on a pronounced rostral curvature causing the sulcus to reorient in a longitudinal manner, with the termination pointing towards the caudal end of the endocast (Connolly 1950: figures 53, 62, 65, 69). In comparison, *L* in *Pan* tends to have a less pronounced rostral curvature (Connolly 1950: figures 80–84; Falk et al. 2018: figures S1, S4, S8). As in *Gorilla* and *Pongo, L* in RUD 200 is especially pronounced, displaying a strong rostral curvature (Figure 1). In RUD 77, *L*, while fainter, still presents evidence of a strong curve (Figure 3). Further, as noted above, *scp* in *Rudapithecus* is more like *Gorilla*/*Pongo* in that the sulcus is large, prominent, and branching sulcus originating on *s* caudal to *c* (e.g., Figures 4A, S3, S23). In *Pan scp* can be reduced (although not universally, Falk et al. 2018: figure S7; see also Figure 4C here) relative to *Gorilla*/*Pongo* and often is only represented by a small notch branching off *s* (Connolly 1950: figures 82–84; Figures S12, S18 here). Lastly, a diagonal sulcus (*d*) is clearly visible in at least one hemisphere in 70% of the *Pan* endocasts studied here, whereas in *Gorilla*/*Pongo* and *Rudapithecus* endocasts *d* is absent. This alone is not conclusive evidence of its absence in *Rudapithecus* (given the small sample), but the frequency of *d* in *Pan* endocasts (14/20, 10 endocasts with two hemispheres analyzed, all frequencies in Table S2), the absence of *d* in *Rudapithecus* is notable.

Though the sulcus patterning in *Rudapithecus* shares attributes with *Gorilla/Pongo* not found in *Pan*, there are additional subtle similarities with *Gorilla*. We were able to identify several sulci on the *Rudapithecus* endocasts that are commonly found in *Gorilla* endocasts but less frequently in those of *Pongo. L* is present in both *Rudapithecus* endocasts and very common in our sample of *Gorilla* (95%), but less in *Pongo* (65%). *Pongo* endocasts tend to be smoother in the superior parietal lobe, which contributes to a lower frequency of sulci being observable in this area. Additionally, the *Rudapithecus* endocasts have clear impressions of *pts* and *ip* on the superior parietal lobe. We were able to identify *pts* in 70% of *Gorilla* endocasts compared with only 15% of *Pongo* endocasts and *ip* in 75% of *Gorilla* vs. 40% of *Pongo* endocasts. Here, we have also observed that the frontal lobe exhibits subtle trends in variation that may provide evidence distinguishing the brains (and endocasts) of *Gorilla* and *Pongo* that have implications for our interpretation of *Rudapithecus*. The anterior termination of *fs* in *Gorilla* is often more rostrally positioned on the dorsal aspect of the frontal lobe (Connolly 1950: figures 67, 74, 76, see also figure 72; Figures 4A, S1, S3 here) than in *Pongo* (Connolly 1950: figures 53, 55–57; Figures 4B, S22, S28 here). In the placement of *fs*, *Rudapithecus* is more *Gorilla*‐like (only visible on RUD 200; Figure 1). Lastly, it is extremely common for *r* in *Pongo* to be extremely rostrally positioned and is almost aligned with the curvature of the endocast, running superior inferiorly at the rostral tip of the frontal lobe (Connolly 1950: figures 53, 56, 57, 59; Figures 4B, S22, S23, S24 here). Conversely, while *r* in *Gorilla* can be oriented inferiorly (Connolly 1950: 65, 72), *r* can also be more inferiorly positioned and run longitudinally for the length of the sulcus to the rostral tip of the frontal lobe more often in *Gorilla* (Connolly 1950: figures 67, 76; Figures 4A, S4, S6 here) than in *Pongo* (Connolly 1950: figures 67, 76; figures 54, 55, 59g; Figures S25, S26 here). In *Rudapithecus*, despite preserving only a partial *r* on both endocasts, the rostral termination is visible and does not reflect the superior–inferior orientation common in *Pongo* but is rather oriented longitudinally in an orientation that is a more common variation in *Gorilla* (Figures 1 and 3). Furthermore, it is notable that the rostral positioning of the anterior termination of *fs* and longitudinal orientation of *r* is seen commonly in the brains of *Pan* as well (Connolly 1950: figures 80–84; Falk et al. 2018: S1, S2, S8), which may suggest these are traits shared by *Rudapithecus* and African apes (see Section 5).

## 5 Discussion

The description of the *Rudapithecus* sulcal pattern here is largely consistent with Begun and Kordos (2004) in their preliminary analysis of the frontal lobe on the *Rudapithecus* endocast. In their analysis, *c*, *arc*, and *r* were identified on both endocasts in addition to two impressions that “likely correspond” to *pcs* and *fs* (Begun and Kordos 2004). In the present analysis we were able to identify a sulcus (*fo*) in RUD 200 not noted by Begun and Kordos (2004). We were unable to confirm the presence of *arc* on RUD 200 (contra Begun and Kordos 2004). On RUD 77, we were not able to confirm the presence of *pcs* (Begun and Kordos 2004) on either side of the endocast.

The absence of sulci on an endocast is a common issue in paleoneurology. Sulci are spaces in the cortex that separate the gyri and are not technically brain surface attributes. As such, sulci may or may not leave impressions on the endocranial surface (Neubauer 2014), making it difficult to determine if some sulci were not present on the brain of a fossil taxon or simply not preserved on rare endocasts (Radinsky 1972; Tobias 1967; Tobias 1987). The meninges and cerebrospinal fluid intervene between the surface of the brain and the skull, so that the endocast is essentially a blurry replica of the original surface of the brain (Neubauer 2014; Radinsky 1972; Tobias 1987). There is also a great deal of variation in the recoverability of morphological detail on endocasts within a given taxon (Radinsky 1972; Tobias 1967; Tobias 1987). It follows that interpretation of endocasts based on the absence of sulci may be problematic. Radinsky (1974) and Falk (1983) interpreted the absence of (*fs*) on the endocast of *Ekembo* (KNM‐RU 7290) as primitive. Did the brain of *Ekembo* actually lack this sulcus or is it simply not preserved on the single known endocast? It is important that any interpretations based on sulcal absence in fossil taxa are made cautiously because even prominent sulci with a high detection rate are not universally detectable on the hominoid endocasts studied here (see Table S2). However, when sample size allows, the frequency of occurrence of a sulcus in extant taxa allows for at least the suggestion that its presence or absence in a fossil may be significant. It is on this basis that we argue that the sulci we identified in *Rudapithecus* that are found more frequently in *Gorilla* endocasts than *Pongo* endocasts may reflect actual phylogenetic affinities (see below).

Some recent studies on *Rudapithecus* have noted some similarities with hylobatids, particularly in the morphology of its pelvis and endocast (Gunz et al. 2020; Ward et al. 2019). Gunz et al. (2020) analyzed a virtual reconstruction of RUD 200 using 3DGM on the shape of both its skull and endocast (see Figure 2). They conclude that RUD 200 most closely resembles African apes in overall skull shape and *Hylobates* in endocranial shape but did not provide an interpretation of the sulcal morphology (Gunz et al. 2020). Nevertheless, the de‐coupling of endocranial and external cranial morphology in RUD 200 is intriguing. Gunz et al. (2020) concluded that while the *Rudapithecus* endocast provides evidence of a great ape level of encephalization, it does not show evidence of a great ape level of brain reorganization, based on the overall surface morphology of the endocast. It should be emphasized that the *Rudapithecus* endocast compared with extant hominoids was virtually reconstructed, with significant mirror imaging to infer the morphology of missing areas, including nearly all of the basicranial portions (inferior occipital, temporal, and frontal lobes).

Our interpretations of the sulcal pattern on the *Rudapithecus* endocast do not indicate a hylobatid‐like sulcal pattern but rather a clear hominid sulcal pattern (i.e., generally more convoluted than hylobatids, separate hook‐shaped *arc*, split ascending tract of the superior temporal sulcus), with some hominine affinities (i.e., greater detection of sulci on endocasts in the superior parietal region, rostrally positioned anterior termination of *fs*, longitudinally oriented *r*). If *Rudapithecus* is indeed more like African apes than *Pongo* in cranial and endocranial morphology (Begun 1994, 2005, 2009, 2010, 2015; Begun 2001; Begun and Kordos 2004; Begun et al. 1997, 2008, 2012; Kordos and Begun 1997, 2001; this study), the primitive overall endocranial shape present in *Rudapithecus* (Gunz et al. 2020) could mean that the shape observed in *Pan*, *Gorilla*, and *Pongo* may have evolved independently. This de‐coupling of sulcal patterning with overall endocranial shape suggests a process of brain evolution in which smaller scale reorganization, based on sulcal pattern modifications, precedes evidence of reorganization as reflected in overall endocranial shape. Begun and Kordos (2004) suggested a de‐coupling of encephalization and brain morphology based on their analysis of the *Rudapithecus* endocasts, finding an absolute and relative brain size in *Rudapithecus* within the range of variation of great apes (confirmed in Gunz et al. 2020), while the morphology of the endocast, particularly the shape of the frontal lobes, appeared more primitive (see also Gunz et al. 2020). Interestingly, decoupling of small‐scale organization and large‐scale morphological changes (i.e., overall shape, size/relative size) is also observed in cercopithecoid brain evolution. The endocast of *Victoriapithecus* exhibits a derived sulcal pattern like that of extant cercopithecoids while lacking the increased brain size of extant taxa (Gonzales et al. 2015). Here, we show that *Rudapithecus* exhibits a relatively derived, great ape‐like sulcal pattern alongside an overall endocranial shape that retains a primitive morphology. It is unknown how these apparently decoupled variables relate to biology, in particular, cognition. It does seem likely, however, given the metabolic expense of brain tissue, that encephalization probably attests to a selective advantage of some sort, probably related to social or ecological cognition (Begun 2024). Why the overall shape of the endocast of RUD 200 should fall closer to hylobatids while the sulcal pattern is clearly hominid, both attributes presumably reflecting cerebral reorganization, remains to be explained. It may be that reorganization occurs first at the sulcal level before being visible in overall morphology.

### 5.1 Phylogenetic and Evolutionary Implications

*Rudapithecus* is generally recognized as either a stem hominine (Begun 1994, 2005, 2009, 2010, 2015; Begun 2001; Begun et al. 1997, 2008, 2012; Kordos and Begun 1997, 2001, 2002; Sevim‐Erol et al. 2023) or stem hominid (Alba 2012; Alba et al. 2015; Harrison 2010; Pugh 2022). In two comprehensive cladistic analyses, a hominine *Rudapithecus* is among several most parsimonious hypotheses presented (Nengo et al. 2017; Pugh 2022), though in the latter it is not the preferred hypothesis. The results of this study are consistent with all previous recent research in finding overwhelming evidence of the great ape (hominid) affinities of *Rudapithecus*. However, the potential African ape affinities of *Rudapithecus* based on endocast morphology are more difficult to interpret. First, the sulcal pattern on the *Pan* frontal lobe is unique among non‐hominin hominids in its complexity (Connolly 1950: figures 53, 65, 82–84; Falk et al. 2018: figures S6–S8) and has been demonstrated to approach australopithecine levels of complexity in some individuals (e.g., *fm* appearing as distinct branches in *Pan* as in *Australopithecus*/*Homo*; see Falk et al. 2018). The increased complexity of the *Pan* sulcal pattern relative to the other non‐hominin hominids was also evident here (see comparisons above). Given the trend towards increased sulcal complexity in hominoid brain evolution (Begun and Kordos 2004; Falk 1983; Falk et al. 2018; Ponce De León et al. 2021; Radinsky 1973, 1974, 1975; Rilling and Insel 1999; Simons 1993; Simons et al. 2007), we interpret the relatively complex sulcal pattern on the *Pan* endocast as a derived trait that more closely approaches the highly convoluted hominin condition than the other taxa included here (Falk et al. 2018; Ponce De León et al. 2021; Rilling and Insel 1999). Despite being clearly primitive relative to *Pan*, the *Rudapithecus* sulcal pattern shares several similarities with *Gorilla* (to the exclusion of *Pongo*; see comparison above) that provide support for both the findings of Gunz et al. (2020) in their analysis of external cranial morphology and the hypothesis of hominine (African ape and human) affinities in *Rudapithecus*.

Comparison of the endocasts of *Aegyptopithecus*, *Ekembo*, *Rudapithecus*, and extant hominoids reveals a clear evolutionary trend towards larger brain size and greater sulcal complexity (Begun 2024; Begun and Kordos 2004; Falk 1983; Falk et al. 2018; Gunz et al. 2020; Kordos and Begun 1998, 2001, 2002; Radinsky 1973, 1974, 1975; Simons 1993; Simons et al. 2007; Walker et al. 1983; Figures S1–S40). The shared attributes of *Rudapithecus*, *Gorilla*, and *Pongo* endocasts and the greater degree of complexity of the sulcal pattern of *Pan* suggest that the shared attributes may reflect the primitive condition for hominids, retained in *Rudapithecus, Gorilla*, and *Pongo*. The greater degree of complexity in *Pan* may be a synapomorphy of the *Pan‐Homo* clade, while the subtle similarities shared uniquely with African apes (primarily *Gorilla*) provide some support for the hominine status of *Rudapithecus*. The polarity of these theoretical character state transformations in hominids is only understandable with consideration of the morphology of fossil endocasts in tandem with other morphological and genetic evidence.

## 6 Conclusion

The endocasts of *Rudapithecus hungaricus* preserve many sulcal impressions. The sulcal pattern on the *Rudapithecus* endocasts is unlike that observed in extant hylobatids and resembles the general sulcal pattern present in the endocasts of extant hominids, especially *Gorilla* and *Pongo*. The *Rudapithecus* endocasts do not possess the complexity present in the endocasts of *Pan* and hominins. However, the sulcal pattern identified in the *Rudapithecus* endocasts displays affinities with *Gorilla* that distinguished its sulcal pattern from that observed in *Pongo*. These results are consistent with the hominine status of *Rudapithecus* (e.g., Begun 2015). Whether or not *Rudapithecus* and other dryopithecins are hominines or stem hominids (e.g., Pugh 2022; Sevim‐Erol et al. 2023), the morphology of its endocasts is informative of an understudied, yet key intermediate stage in the evolution of the hominid brain occurring in the late Miocene.

## Author Contributions

**Griffin A. Assance:** conceptualization, investigation, writing – original draft, writing – review and editing, visualization, validation, methodology, project administration. **Mary T. Silcox:** investigation, writing – review and editing, supervision, resources, funding acquisition. **David R. Begun:** investigation, funding acquisition, writing – review and editing, data curation, supervision, resources.

## Funding

This work was supported by the Natural Sciences and Engineering Research Council of Canada and Beausoleil First Nation.

## Supporting information


**Data S1:** ajpa70218‐sup‐0001‐Figures.docx.

**Table S1:** Comparative sample practical information.

**Table S2:** Sulci identification data.

## Data Availability

All endocasts will be made available on MorphoSource (pending upload). The information on data acquisition for the raw CT files (including MorphoSource links) of the extant sample is available in Table S1.

## Endnotes

^1^
RUD numbers are field number assigned as specimens are recovered and identified in the field, starting with the first publications of the Rudabánya primates (e.g., Kretzoi 1975). We continue this tradition here, to facilitate reference to previous research. Both RUD 77 and RUD 200 have official inventory numbers of the Department of Collections of the Szabályozott Tevékenységek Felügyeleti Hatósága (SZTFH), or the Regulated Activities Supervisory Authority, in English. For RUD 77 the inventory number is V.2015.209. RUD 200 was originally assigned multiple field numbers as specimens separately came to light before it was recognized that they all belonged to the same specimen (i.e., RUD 197 [right upper lateral incisor] = V.2015.329; RUD 198 [left upper canine] = V.2015.330; RUD 199 [right upper central incisor] = V.2015.331; RUD 200 [cranium] = V.2015.332; RUD 207 [left upper lateral incisor] = V.2015.339; RUD 212 [mandible with complete dentition] = V.2015.3440). For brevity we refer to all of them in the text as RUD 200.
