# Supplementary material for: Comparative Sulcal Morphology of the Late Miocene Fossil Ape, Rudapithecus hungaricus
Source: Am J Biol Anthropol. 2026 Feb 23;189(2):e70218. doi: 10.1002/ajpa.70218 (PMC12929042; doi:10.1002/ajpa.70218)
Supplement: Supplementary file 1 — Data S1: ajpa70218‐sup‐0001‐Figures.docx. [file AJPA-189-e70218-s002.docx]

**Figure S1: Female *G. gorilla* (AGH 04) endocast with labelled sulcal patterning. A) Right lateral view, B) Left lateral view, C) Dorsal view, D) Rostral view, E) Caudal view.**

**A)**

**B)**

**C)**

**D)**

**E)**

**Figure S2: Female *G. gorilla* (AGH 12) endocast with labelled sulcal patterning. A) Right lateral view, B) Left lateral view, C) Dorsal view, D) Rostral view, E) Caudal view.**

**A)**

**B)**

**C)**

**D)**

**E)**

**Figure S3: Female *G. gorilla* (AGH 21) endocast with labelled sulcal patterning. A) Right lateral view, B) Left lateral view, C) Dorsal view, D) Rostral view, E) Caudal view.**

**A)**

**B)**

**C)**

**D)**

**E)**

**Figure S4: Female *G. gorilla* (AMNH 99-9687) endocast with labelled sulcal patterning. A) Right lateral view, B) Left lateral view, C) Dorsal view, D) Rostral view, E) Caudal view.**

**A)**

**B)**

**C)**

**D)**

**E)**

**Figure S5: Female *G. gorilla* (MCZ 37265) endocast with labelled sulcal patterning. A) Right lateral view, B) Left lateral view, C) Dorsal view, D) Rostral view, E) Caudal view.**

**A)**

**B)**

**C)**

**D)**

**E)**

**Figure S6: Male *G. gorilla* (AGH 08) endocast with labelled sulcal patterning. A) Right lateral view, B) Left lateral view, C) Dorsal view, D) Rostral view, E) Caudal view.**

**A)**

**B)**

**C)**

**D)**

**E)**

**Figure S7: Male *G. gorilla* (AGH 09) endocast with labelled sulcal patterning. A) Right lateral view, B) Left lateral view, C) Dorsal view, D) Rostral view, E) Caudal view.**

**A)**

**B)**

**C)**

**D)**

**E)**

**Figure S8: Male *G. gorilla* (AGH 17) endocast with labelled sulcal patterning. A) Right lateral view, B) Left lateral view, C) Dorsal view, D) Rostral view, E) Caudal view.**

**A)**

**B)**

**C)**

**D)**

**E)**

**Figure S9: Male *G. gorilla* (AGH 19) endocast with labelled sulcal patterning. A) Right lateral view, B) Left lateral view, C) Dorsal view, D) Rostral view, E) Caudal view.**

**A)**

**B)**

**C)**

**D)**

**E)**

**Figure S10: Male *G. gorilla* (AGH 23) endocast with labelled sulcal patterning. A) Right lateral view, B) Left lateral view, C) Dorsal view, D) Rostral view, E) Caudal view.**

**A)**

**B)**

**C)**

**D)**

**E)**

**Figure S11: Female *P. troglodytes* (MCZ 17702) endocast with labelled sulcal patterning. A) Right lateral view, B) Left lateral view, C) Dorsal view, D) Rostral view, E) Caudal view.**

**A)**

**B)**

**C)**

**D)**

**E)**

**Figure S12: Female *P. troglodytes* (MCZ 26849) endocast with labelled sulcal patterning. A) Right lateral view, B) Left lateral view, C) Dorsal view, D) Rostral view, E) Caudal view.**

**A)**

**B)**

**C)**

**D)**

**E)**

**Figure S13: Female *P. troglodytes* (USNM 84655) endocast with labelled sulcal patterning. A) Right lateral view, B) Left lateral view, C) Dorsal view, D) Rostral view, E) Caudal view.**

**A)**

**B)**

**C)**

**D)**

**E)**

**Figure S14: Female *P. troglodytes* (USNM 175707) endocast with labelled sulcal patterning. A) Right lateral view, B) Left lateral view, C) Dorsal view, D) Rostral view, E) Caudal view.**

**A)**

**B)**

**C)**

**D)**

**E)**

**Figure S15: Female *P. troglodytes* (USNM 176229) endocast with labelled sulcal patterning. A) Right lateral view, B) Left lateral view, C) Dorsal view, D) Rostral view, E) Caudal view.**

**A)**

**B)**

**C)**

**D)**

**E)**

**Figure S16: Male *P. troglodytes* (AMNH 51204) endocast with labelled sulcal patterning. A) Right lateral view, B) Left lateral view, C) Dorsal view, D) Rostral view, E) Caudal view.**

**A)**

**B)**

**C)**

**D)**

**E)**

**Figure S17: Male *P. troglodytes* (AMNH 167342) endocast with labelled sulcal patterning. A) Right lateral view, B) Left lateral view, C) Dorsal view, D) Rostral view, E) Caudal view.**

**A)**

**B)**

**C)**

**D)**

**E)**

**Figure S18: Male *P. troglodytes* (AMNH 167344) endocast with labelled sulcal patterning. A) Right lateral view, B) Left lateral view, C) Dorsal view, D) Rostral view, E) Caudal view.**

**A)**

**B)**

**C)**

**D)**

**E)**

**Figure S19: Male *P. troglodytes* (MCZ BOM 6244) endocast with labelled sulcal patterning. A) Right lateral view, B) Left lateral view, C) Dorsal view, D) Rostral view, E) Caudal view.**

**A)**

**B)**

**C)**

**D)**

**E)**

**Figure S20: Male *P. troglodytes* (USNM 220327) endocast with labelled sulcal patterning. A) Right lateral view, B) Left lateral view, C) Dorsal view, D) Rostral view, E) Caudal view.**

**A)**

**B)**

**C)**

**D)**

**E)**

**Figure S21: Female *P. abelii* (DMNS 10464) endocast with labelled sulcal patterning. A) Right lateral view, B) Left lateral view, C) Dorsal view, D) Rostral view, E) Caudal view.**

**A)**

**B)**

**C)**

**D)**

**E)**

**Figure S22: Female *P. pygmaeus* (MCZ 31518) endocast with labelled sulcal patterning. A) Right lateral view, B) Left lateral view, C) Dorsal view, D) Rostral view, E) Caudal view.**

**A)**

**B)**

**C)**

**D)**

**E)**

**Figure S23: Female *P. pygmaeus* (MCZ 37519) endocast with labelled sulcal patterning. A) Right lateral view, B) Left lateral view, C) Dorsal view, D) Rostral view, E) Caudal view.**

**A)**

**B)**

**C)**

**D)**

**E)**

**Figure S24: Female *P. pygmaeus* (USNM 142185) endocast with labelled sulcal patterning. A) Right lateral view, B) Left lateral view, C) Dorsal view, D) Rostral view, E) Caudal view.**

**A)**

**B)**

**C)**

**D)**

**E)**

**Figure S25: Female *P. pygmaeus* (USNM 142187) endocast with labelled sulcal patterning. A) Right lateral view, B) Left lateral view, C) Dorsal view, D) Rostral view, E) Caudal view.**

**A)**

**B)**

**C)**

**D)**

**E)**

**Figure S26: Male *P. abelii* (USNM 143588) endocast with labelled sulcal patterning. A) Right lateral view, B) Left lateral view, C) Dorsal view, D) Rostral view, E) Caudal view.**

**A)**

**B)**

**C)**

**D)**

**E)**

**Figure S27: Male *P. abelii* (USNM 143590) endocast with labelled sulcal patterning. A) Right lateral view, B) Left lateral view, C) Dorsal view, D) Rostral view, E) Caudal view.**

**A)**

**B)**

**C)**

**D)**

**E)**

**Figure S28: Male *P. abelii* (USNM 143594) endocast with labelled sulcal patterning. A) Right lateral view, B) Left lateral view, C) Dorsal view, D) Rostral view, E) Caudal view.**

**A)**

**B)**

**C)**

**D)**

**E)**

**Figure S29: Male *P. pygmaeus* (USNM 142181) endocast with labelled sulcal patterning. A) Right lateral view, B) Left lateral view, C) Dorsal view, D) Rostral view, E) Caudal view.**

**A)**

**B)**

**C)**

**D)**

**E)**

**Figure S30: Male *P. pygmaeus* (USNM 142184) endocast with labelled sulcal patterning. A) Right lateral view, B) Left lateral view, C) Dorsal view, D) Rostral view, E) Caudal view.**

**A)**

**B)**

**C)**

**D)**

**E)**

**Figure S31: Female *H. lar* (MCZ 41440) endocast with labelled sulcal patterning. A) Right lateral view, B) Left lateral view, C) Dorsal view, D) Rostral view, E) Caudal view.**

**A)**

**B)**

**C)**

**D)**

**E)**

**Figure S32: Female *H. lar* (MCZ 41463) endocast with labelled sulcal patterning. A) Right lateral view, B) Left lateral view, C) Dorsal view, D) Rostral view, E) Caudal view.**

**A)**

**B)**

**C)**

**D)**

**E)**

**Figure S33: Female *H. lar* (MCZ 41469) endocast with labelled sulcal patterning. A) Right lateral view, B) Left lateral view, C) Dorsal view, D) Rostral view, E) Caudal view.**

**A)**

**B)**

**C)**

**D)**

**E)**

**Figure S34: Female *H. lar* (MCZ 41493) endocast with labelled sulcal patterning. A) Right lateral view, B) Left lateral view, C) Dorsal view, D) Rostral view, E) Caudal view.**

**A)**

**B)**

**C)**

**D)**

**E)**

**Figure S35: Female *Ho. leuconednys* (AMNH 201743) endocast with labelled sulcal patterning. A) Right lateral view, B) Left lateral view, C) Dorsal view, D) Rostral view, E) Caudal view.**

**A)**

**B)**

**C)**

**D)**

**E)**

**Figure S36: Male *H. lar* (USNM 111970) endocast with labelled sulcal patterning. A) Right lateral view, B) Left lateral view, C) Dorsal view, D) Rostral view, E) Caudal view.**

**A)**

**B)**

**C)**

**D)**

**E)**

**Figure S37: Male *H. lar* (USNM 111989) endocast with labelled sulcal patterning. A) Right lateral view, B) Left lateral view, C) Dorsal view, D) Rostral view, E) Caudal view.**

**A)**

**B)**

**C)**

**D)**

**E)**

**Figure S38: Male *H. lar* (USNM 143564) endocast with labelled sulcal patterning. A) Right lateral view, B) Left lateral view, C) Dorsal view, D) Rostral view, E) Caudal view.**

**A)**

**B)**

**C)**

**D)**

**E)**

**Figure S39: Male *Ho. hoolock* (AMNH 112720) endocast with labelled sulcal patterning. A) Right lateral view, B) Left lateral view, C) Dorsal view, D) Rostral view, E) Caudal view.**

**A)**

**B)**

**C)**

**D)**

**E)**

**Figure S40: Male *Ho. leuconednys* (AMNH 201742) endocast with labelled sulcal patterning. A) Right lateral view, B) Left lateral view, C) Dorsal view, D) Rostral view, E) Caudal view.**

**A)**

**B)**

**C)**

**D)**

**E)**
